# Applications and perspectives of the use of ultrasonography for wildlife andrology: a review

**DOI:** 10.1590/1984-3143-AR2025-0038

**Published:** 2025-10-17

**Authors:** Bruno Galvão de Matos Brito, Radan Elvis Matias de Oliveira, Alexandre Rodrigues Silva

**Affiliations:** 1 Laboratório de Conservação de Germoplasma Animal, Centro de Ciências Agrárias, Universidade Federal Rural do Semi Árido, Mossoró, RN, Brasil; 2 Curso de Medicina Veterinária, Centro de Ciências da Saúde, Universidade de Fortaleza, Fortaleza, CE, Brasil; 3 Universidade Federal Rural do Semi Árido, Mossoró, RN, Brasil

**Keywords:** wildlife, male reproduction, ultrasound, doppler

## Abstract

Application of assisted reproduction techniques are essential for the preservation of endangered species, and ultrasonography has emerged as an interesting tool in this process, allowing noninvasive assessment of reproductive stages and characterization of male gonads. This review provides a compilation on the applications and perspectives of using ultrasonography for investigation of the morphological and functional aspects of the male reproductive tract in wild species. The technique, which has been improved with the use of vascular doppler, allows detailed analysis of blood flow and aids in the selection of individuals for breeding programs. Although there are challenges, such as physiological variations among species and the difficulty for applying ultrasonography to birds and reptiles, advances in imaging technologies, including elastography and doppler, have expanded the possibilities for diagnosis and monitoring reproductive status in various mammals. Ultrasonographic analysis contributes to the assessment of fertility, detection of testicular diseases and the definition of protocols for reproductive management, becoming an important tool in the conservation of wildlife and in the development of more effective assisted reproductive technologies.

## Introduction

Assisted reproduction techniques play a fundamental role in the preservation and perpetuation of wildlife in the natural environment, especially in the case of those that are threatened with extinction. Several protocols involving the manipulation of the reproductive process, such as the collection of male gametes, have been developed with the aim of ensuring the perpetuation of different species (Santos and Silva, 2023). For this purpose, however, knowledge on their physiology is essential for the definition of gamete collection protocols. Although widely applied to domestic animals of economic or emotional relevance, methods that aim to promote reproduction in wild species represent a challenge due to the physiological variations found, even among individuals of the same genus or family (Zelli et al., 2023). Each group has particularities that need to be understood so that more appropriate techniques can be developed.

Male reproductive physiology appears as an essential field of study for reproductive success. Andrology is the area that studies the male reproductive system, including its gonads, leading to the understanding of the mechanisms of male gamete production. Based on this knowledge, it is possible to establish techniques for manipulating these cells, making fertilization and consequently the production of new individuals (Alfaro, 2011). The identification of testicular disorders based on the andrological examination also contributes to the optimization of the male's genetic material and may indicate the selection of viable animals or even the appropriate treatment to reverse the disease in those animals (Pinto et al., 2024). In this context, ultrasound allows the clarification of reproductive physiological characteristics and the diagnosis of pathological changes, being a tool used in the evaluation and reproductive monitoring of males in a non-invasive manner (Santos et al., 2021).

Ultrasonography has been widely used in veterinary medicine since its pioneering application for assessing pregnancy in small ruminants in the United States in the 1960s (Lindahl, 1966). As a non-invasive procedure, this technique allows obtaining important morphological information, including the identification of pathological changes, monitoring the evolution of treatments and reproductive characterization (Lotti and Maggi, 2015). In addition, it has proven to be a valuable tool in research on domestic and wild animals’ reproduction, providing detailed data on physiological aspects. Ultrasonography allows the morphological evaluation of different organs in different stages of the reproductive cycle or reproductive seasons (Dalmazzo and Ferrari, 2019). Characteristics such as echogenicity, echotexture, size, contour and relationship with adjacent organs are analyzed in domestic animals, revealing correlations with the functionality of the gonads (Huang et al., 2021), which can also be observed in wild species (Brook et al., 2000). By generating different echoes according to the histological constitution of the tissue, ultrasound allows its characterization and correlation with tissue physiology (Yamaguchi, 2021). This technology makes it possible, for example, to analyze the histological variations of testicular tissue throughout pre- and post-puberty development (Abdelkhalek et al., 2022).

The most widely used modality in veterinary medicine is B-mode ultrasound, which translates tissue echoes into a two-dimensional image, with different shades of gray, depending on the density of the organs, which facilitates their measurement and analysis directly or with the aid of computerized analysis (Brito et al., 2012). In recent years, ultrasound has undergone significant evolution with the introduction of new parameters, such as vascular Doppler, which allows a detailed analysis of blood flow in the reproductive organs, providing greater accuracy to examinations (Samir et al., 2023). Based on the physics of sound wave laws, doppler ultrasound detects changes in the frequency of the sound wave generated by the movement of blood in the vessels, allowing the evaluation of aspects such as blood flow velocity and hemodynamic index, such as the resistivity index (RI), the pulsatility index (PI) and the systole-diastole ratio (S/D) (Bollwein et al., 2002). This advance has proven to be fundamental in the reproductive characterization of wild animals, facilitating its application in the selection of individuals for breeding programs aimed at the conservation and maintenance of these species (Abdelkhalek et al., 2022). Furthermore, different Doppler modalities have been developed for specific purposes, such as color Doppler for vascular mapping, continuous Doppler, pulsed or spectral Doppler and amplitude Doppler, expanding the analysis possibilities (Bollwein et al., 2002).

Given these technological advances, ultrasonography has emerged as a promising tool for the conservation of wild species, aiding both in the selection of individuals for reproductive programs and in monitoring their reproductive health. Thus, the objective of this review is to compile data from the literature that describe the ultrasonographic aspects of the male reproductive system of wild animals, correlating them with the reproductive capacity of the species, in addition to explore future perspectives for the use of this technique in wildlife conservation.

The review was based on articles from major scientific platforms (e.g., PubMed, Scopus, Web of Science, SciELO, MyLibrary), using keywords such as ultrasound, andrology, and wild species. Studies in multiple languages and without time restrictions were included to ensure a comprehensive and inclusive overview. One challenge encountered was the difficulty in retrieving all relevant literature, as some articles refer only to specific species names without broader terms like “wildlife” or “non-domestic animals,” which can limit search results and require a more refined and manual selection process.

## Challenges for application of ultrasound in wildlife reproduction

In wild animals, the preparation for abdominal ultrasound examination is similar in many aspects to the preparation for domestic animals, with fasting and trichotomy of the area to be scanned being indicated (Hildebrandt and Saragusty, 2015). Fasting may be necessary to minimize artifacts due to the presence of food or gases in the digestive tract that interfere with obtaining adequate images, while trichotomy prevents the formation of air pockets between the fur and the skin, which would cause a reverberation effect, interfering with the image formation. It is worth noting that both fasting and trichotomy must consider physiological aspects of the species, such as type of food, gastrointestinal transit speed, and maintenance of body temperature (O’Grady et al., 1978). For some low studied species, however, such information could be unavailable thus impairing the examination efficiency.

Another important consideration is the feasibility of performing ultrasound examinations in remote locations. Advances in ultrasound technology have made it possible to acquire high-quality images using portable, battery-powered devices (Salimi et al., 2022). While standard batteries may only provide about 1.5 hours of scanning in B-mode, the use of extended batteries (e.g., Mindray Vetus E7 U-bank) can increase operating time to up to 8 hours. However, battery life varies depending on the imaging mode used, such as B-mode, color Doppler, or elastography.

The wide anatomical variety among species is a challenge. In birds, the use of ultrasound is restricted due to anatomical issues. In addition to their reduced size, birds have air sacs in their coelomic cavities that cause the formation of artifacts and make it difficult to visualize the organs. Therefore, ultrasound does not appear to be the most appropriate method for this group of species (Bende et al., 2023). Despite this, some Galliformes species have reduced air sacs and allow adequate ultrasound examination, including their gonads (Gros et al., 2022). This fact demonstrates the need for anatomical characterization of the species to validate the use of ultrasound technique.

The reptiles consist of another group that deserves special attention. The main reason is because there is a huge variety of body conformation, since snakes, lizards and turtles are included in this group. Scales, hooves and plastron are structures that can hinder or prevent the proper passage of ultrasound, limiting the area of access for the examination. In addition, some species of reptiles have air sacs in their coelomic cavities, causing the same interference described for birds, as in some chameleons, lizards and snakes (Gardhouse, 2023).

In addition to anatomical characteristics, another factor that directly interferes with the performance of the ultrasound examination is the temperament of the species. This is because in docile animals, physical restraint may be sufficient for a good ultrasound performance. However, in aggressive individuals or in pain or another condition that prevents physical restraint, sedation may be necessary (Gardhouse, 2023). In river dolphins (*Inia geoffrensis*), physical restraint is sufficient for performing the ultrasound examination (Alves et al., 2012). The same is true for dolphins (*Tursiops truncatus aduncas*), but in these cases the morphometric evaluation becomes more reliable when restraint occurs outside the water, reducing interference from the animal's movements (Brook et al., 2000).

**Figure 1:**
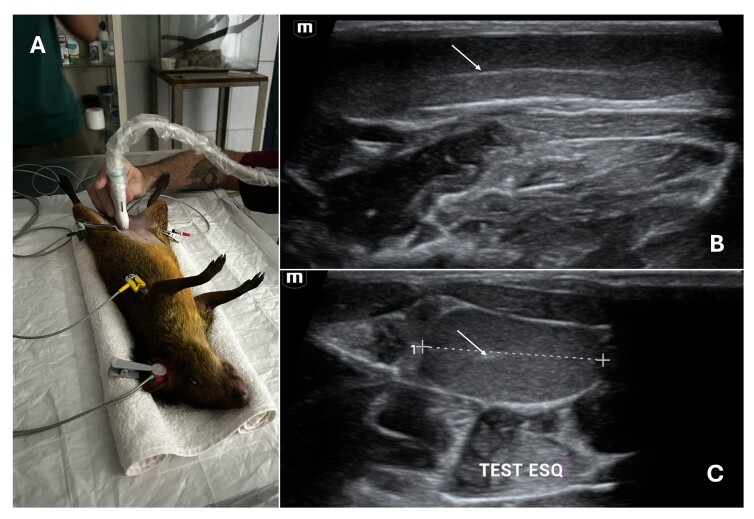
Ultrasound exam of agouti’s male reproductive system (*Dasyprocta leporina*). (A) Animal in dorsal recumbency, sedated and monitored, with ventral transabdominal ultrasound access; (B) B-mode ultrasound image of agouti testis in sagittal section showing the testicular mediastinum as a hyperechogenic line (arrow) and parenchyma with homogeneous echotexture and mixed echogenicity; (C) Agouti testis in cross section with hyperechoic central and circular mediastinum (arrow). Note the elongated shape of the testis in this species, as described in the literature.

For some species, chemical restraint becomes mandatory, ensuring safety for the animal and for professionals (Figure 1A). This is the case of the crab-eating fox (*Cerdocyon thous*), in which Carvalho et al. (2020) used midazolam (0.5 mg/kg) and ketamine (10 mg/kg) prior to the ultrasound examination. For brocket deer (*Mazama gouazobira*), Cunha et al. (2019) used the combination of ketamine (5 to 10 mg/kg) and xylazine (0.5 to 1.5 mg/kg); however, in this case, the team proceeded with electroejaculation, requiring a longer sedation time. For *Dasyprocta leporine* rodents, the ketamine-xylazine combination (35 mg/kg – 5 mg/kg) was also chosen when the electroejaculation procedure was performed (Lima et al., 2023), but there is no data on the doses for sedation in the case of ultrasound examination.

The individual temperament of the animal to be handled must always be taken into consideration, with preference given to protocols with physical restraint and minimal stress. Hildebrandt et al. (1998), for example, reported the use of three different protocols for three elephants of the species *Loxodonta africana*. This difference was due to individuals being in different social situations. One free-living individual needed to be sedated (Etorphine 11.25 mg) and restrained in lateral decubitus. Another captive animal was sedated (xylazine, 800 mg; carfentanil, 0.5 mg) and examined in a stationary position. A third animal did not require chemical sedation. This demonstrates the need for individual assessment to establish restraint protocols, in addition to knowing the dose required for each species.

Despite presenting a great anatomical variety, ultrasound examination in wild animals demonstrates similarities with domestic species, since the echogenicity and echotexture of the organs do not usually vary greatly (O’Grady et al., 1978). However, studies related to the species should be conducted to characterize the ultrasound findings, ensuring an adequate ultrasound evaluation. Below we present some results obtained through ultrasound evaluation of the male reproductive system of different species of wild animals.

## Male gonads morphological ultrasound characteristics

The ultrasound image of the testicles tends to be homogeneous between species. Its echogenic appearance with homogeneous echotexture contrasts with a hyperechoic central region that represents the testicular mediastinum composed of the rete testis (Ali et al., 2011; Bartlewski et al., 2017; Ribeiro et al., 2017). This could be seen in Figures 1B and 1C, showing de agouti’s testicle ultrasound image. Variations between species occur mainly in relation to the position of the rete testis, which may be more peripheral as in horses, in addition to changes in echogenicity between the prepubertal and pubertal stages (Abdelkhalek et al., 2022). Table 1 lists the testicles ultrasound characteristics of different wild species.

**Table 1:** Ultrasound characteristics of the testis and epididymis of different wild species.

	**Specie**	**Testicle**	**Epididymis**	**Authors**
Aquatic	**Freshwater Dolphin** *(Inia Geoffrensis)*	Hyperechogenic testicular mediastinum; uniform heterogeneous pattern with nodules in sexually mature animals	Triangular head, cranial end of the testicle, iso- or hyperechogenic in relation to the testicle. Medial body. Hyperechogenic tail.	Alves et al. (2012)
	**Bottlenose dolphin** *(Tursiops truncatus aduncas)*	Hyperechoic testicular mediastinum in the form of a central line extending along the entire length; parenchyma iso- or hyperechoic compared to the adjacent sublombar muscle. Lobulations are present, especially in older animals. Echogenicity increases with age.	Iso- or hyperechogenic to the testicle. Body related to the lateral face of the testicle and triangular cross-section. Hypoechogenic tail with indistinguishable tubular pattern.	Brook et al. (2000)
	**Atlantic salmon** *(Salmo salar)*	Black, oval or circular structure near the intestine and kidneys. Not seen in all reproductive stages.	x	Næve et al. (2019)
	**Yangtze finless porpoise** *(Neophocaena phocaenoides asiaeorientalis)*	Parenchyma with variable echogenic pattern between individuals. Sexually mature individuals with echogenic patterns are considered strong. Hyperechogenic mediastinum along the entire testicular length.	x	Wu et al. (2010)
Amphibian	**Proteus** *(Proteus anguinus)*	homogeneous echotexture and intermediate echogenicity.	x	Holtze et al. (2017)
Testudine	**Loggerhead Sea Turtles** *(Caretta caretta)*	Uniform echotexture. Hyperechogenic in relation to adjacent kidneys.	Numerous anechogenic and tubular structures caudal to the testis.	Pease et al. (2010)
Carnivore	**Palawan Bearcats** *(Arctictis binturong whitei)*	Grossly hypoechogenic parenchyma surrounded by a thin echogenic layer representing the tunica albuginea.	x	Lajara et al. (2021)
	**Meerkats** *(Suricata suricatta)*	Testicular parenchyma: Fine texture, homogeneous, medium echogenicity with hyperechogenic surrounding structure Mediastinum: Not evident; slight hyperechogenic central line parallel to long axis	Partially visible, elongated hypoechogenic structure with rounded edges at caudoventral surface	Silvatti et al. (2020)
Ursid	**Koala** *(Phascolarctos cinereus)*	Parenchyma of homogeneous echogenicity, without apparent mediastinum, surrounded by hyperechoic tunica albuginea.	Hypo- and hyperechogenic regions. Only in the sagittal section due to its reduced size and proximity to the testicle	Larkin et al. (2018)
	**Crab-eating fox** *(Cerdocyon thous)*	Central and hyperechogenic testicular mediastinum; parenchyma with homogeneous echotexture. Similar to dogs, bulls and cats.	x-	Carvalho et al. (2020)
Ruminant	**Gray Brocket Deers** *(Mazama gouazoubira)*	Smooth echotexture, hyperechogenic longitudinal linear mediastinum as well as the tunica albuginea.	Hypoechogenic tail of the epididymis is also surrounded by the tunica albuginea.	Cunha et al. (2019)
Large Mammals	**Elephant** *(Loxodonta Africana e Elephas maximus)*	Hypoechogenic and homogeneous parenchyma, divided by moderately echogenic septa. Mediastinum difficult to access from the intra-abdominal position, ex situ appears as an irregular and hyperechogenic structure.	Epididymis is much smaller than the testis. Differentiation of the two structures may be difficult by ultrasound. Slightly hypoechoic, with the lumen of the epididymal tubules anechoic. There is no difference between head, body and tail.	Hildebrandt et al. (1998)
	**Elephant** *(Loxodonta Africana e Elephas maximus)*	Differences in the echogenicity of the testicle of sexually active (hypoechogenic) and inactive (hyperechogenic) males, due to testicular vascularization.	x	Hildebrandt et al. (2000)
	**Camel** *(Camelus dromedarius)*	Parenchyma with homogeneous echotexture and central echogenic area representing the testicular network.	x	Waheed et al. (2014)
	**Sumatran Rhinoceros** *(Dicerorhinus sumatrensis)*	Homogeneous echotexture with central hyperechogenic area representing the mediastinum.	Hypoechogenic in relation to the testicle, head and tail surrounded by anechoic structures.	Zahari et al. (2002)
	**Rothschild’s giraffes** (*Giraffa camelopardalis rotschildi*)	Moderate parenchyma echogenicity, hyperechogenic mediastinum (5.5mm) with fine peripheral septulae.	x	Lueders et al. (2009)
Suids	**Collared Peccaries** *(Pecari tajacu)*	Echogenic parenchyma with homogeneous echotexture and presence of hyperechogenic central mediastinum	x	Peixoto et al. (2015)
	**Aardvark** *(Orycteropus Afer)*	Homogeneous parenchyma of medium echogenicity with multiple hyperechogenic foci. Central and hyperechogenic mediastinum.	Heterogeneous with several anechogenic structures.	Wojick et al. (2018)
Primates	**Marmoset** *(Saguinus ursulus)*	Homogeneous echotexture with medium to coarse granulation, and a medium to a high degree of echogenicity. The testicular mediastinum was observed in only one animal, discreetly.	x	Borges et al. (2020)
	**St. Kitts vervet monkey** *(Chlorocebus sabaeus)*	Fine echotexture. Centrally, hyperechogenic mediastinal testis was visualized in all males.	Hypoechogenic in relation to the testicle.	Amory et al. (2012)

Specialized software has been developed for quantitative analysis of ultrasonographic images. Ecotext (Humeco, Spain) enables characterization of testicular parenchyma at both macroscopic and microscopic levels, though species-specific calibration is required for accurate analysis (Araújo et al., 2021). The histological composition of testicular tissue is responsible for its ultrasound appearance (Brito et al., 2012). Thus, depending on the tissue organization of the species, the ultrasound image obtained will be different. To interpretate this image, it is essential to know the normal appearance, since these differences in organization can lead to erroneous conclusions. The testicular mediastinum is present in the image as a hyperechogenic area in most individuals (Figure 1), but this characteristic does not occur in some species, such as the koala (*Phascolarctos cinereus*) (Larkin et al., 2018).

Specific characteristics are also related to their echotexture (Figure 1), varying slightly between species (Lajara et al., 2021; Larkin et al., 2018). The characterization of the echogenicity of the species must consider the underlying organs, since it is a comparative and subjective data (Pease et al., 2010). In species where the male gonad is located within the abdominal cavity, the echogenicity comparison with the sublumbar muscles can be used, as is the case of *Tursiops truncatus aduncas* dolphins. In this specie, due to the proximity of the testicle located caudal to the kidney, it is possible to compare the two structures in a single image. Since the image produced by muscles does not usually vary between species, this comparative data becomes interesting for characterization (Brook et al., 2000).

It is important to note that variations in normality are found between species of the same group. In non-human primates, for example, it is possible to find testicles with medium to coarse echotexture, such as in the species *Saguinus ursulus* (Borges et al., 2020), while others have a smoother echotexture, such as in *Chlorocebus sabaeus* (Amory et al., 2012). Differences in the appearance of the testicular mediastinum between species are also reported. (Table 1).

The echogenicity of testicular tissue can vary from hypo to hyperechogenic, but it is important to verify the reproductive status of that individual before taking conclusions. Changes in echogenicity can occur due to diseases, but are also present in physiological situations involving sexual maturity, as described for dolphins - *Tursiops truncatus aduncas* (Brook et al., 2000), elephants - *Loxodonta africana* and *Elephas maximus* (Hildebrandt et al., 2000), Chinese smooth-mouth dolphin - *Neophocaena phocaenoides asiaeorientalis* (Wu et al., 2010) and river dolphin - *Inia Geoffrensis* (Alves et al., 2012) (Table 1). In other cases, the normal characteristic of the testicle of the species may resemble pathological images of other species, as in the case of the aardvark (*Orycteropus afer*) which naturally presents multiple hyperechogenic foci in its parenchyma (Wojick et al., 2018).

The B-mode testicular ultrasound is important for investigating the echogenicity and echotexture of the testicular parenchyma, which may indicate the reproductive capacity of the animal being evaluated. In bulls, increased echogenicity in older animals is a described characteristic and should be correlated with semen quality (Munywoki et al., 2024). The method can also be used to access testicular morphometry data, presenting values very close to the real ones using a minimally invasive method (Peixoto et al., 2015). In echidnas (Johnston et al., 2007) and jaguars (Requena et al., 2023), positive correlations were found between testicular size measured on ultrasound and semen quality. In Neotropical boid snakes, testis size varied according to the reproductive season (Garcia and Almeida‐Santos, 2022). Although linear measurements present an excellent correlation, it should be considered that ultrasound produces two-dimensional images and therefore it is necessary to apply mathematical formulas to predict the volume of the structures. The species, weight and method of collecting morphometric data should be considered for analysis (Homola et al., 2025).

In collared peccaries (*Pecari tajacu*), morphological evaluation of the testis by ultrasonography is an important tool for assessing reproductive capacity, since morphometric measurements of the gonad are related to seminal quality, especially when using the Lambert formula (Peixoto et al., 2015). These findings show that testicular volume should be assessed considering the shape of the testicles of each species.

Structures adjacent to the testicle should also be examined to assess the physiological aspects of the gonad. Data on ultrasound imaging of the epididymis in different species are listed in Table 1. It is possible to assess the epididymis, which appears hypoechogenic in relation to the testicular parenchyma. Moreover, the spermatic cord containing the testicular artery, testicular veins, vas deferens and cremaster muscle can also be assessed in B mode, where the vessels appear as anechoic tubular structures (Zahari et al., 2002). On the other hand, there is a restriction in the assessment of vascular aspects through the B mode ultrasound, since it is not possible to qualitatively assess blood flow to the organ. A mode based on the sound doppler effect was developed to obtain this information.

The evaluation of blood flow in male gonads has been shown to be a good marker for the reproductive capacity of domestic males (Velasco and Ruiz, 2020). Data on blood flow characteristics and its interference in sperm quality and puberty have already been described in dogs, horses, and sheep (Souza et al., 2015; Ortiz-Rodriguez et al., 2017; Elbaz et al., 2019). However, in an experiment with brocket deer (*Mazama gouazoubira*), there was no correlation between the values of the indices obtained with Doppler between the groups with normal fertility and sub fertile or infertile groups (Cunha et al., 2019).

Regarding the identification of pathological processes that can compromise fertility, ultrasound examination can detect deleterious alterations, but normal images do not rule out subfertility. In contrast, certain sonographic alterations, including hyperechogenic spots within the testicular parenchyma, potentially represent senescence-associated fibrosis. Nevertheless, these ultrasonographic changes in white rhinoceros (*Ceratotherium simum simum, C.s. cottoni)* showed no correlation with impaired sperm fertilizing capacity (Hermes et al., 2005). Because it is an accessible method, ultrasound can function as a screening for testicular alterations in animals. A testicular neoplasia was identified in a giant panda that, based on the results of the ultrasound, underwent general anesthesia for other more detailed examinations, such as magnetic resonance imaging, to better characterize the lesion and assist in prescribing the most appropriate treatment (Lord et al., 2020). In dogs, testicular neoplasia is the most common abnormality (Bracco et al., 2023). In wild animals, we do not have sufficient data to indicate a higher prevalence.

For wildlife, the diagnosis of pathological alterations becomes difficult and often occurs during necropsy. However, these findings contribute to the characterization of these diseases in the species and set a precedent for expanding diagnostic studies, as is the case of odontocetes of the species *Phocoena phocoena*. Gregor et al. (2022) describe the postmortem findings of specimens of the species, indicating the occurrence of testicular neoplasia related to Sertoli cells.

## Accessory sex glands ultrasonography

In addition to the male gonads, B-mode allows the evaluation of other structures related to reproduction. The accessory sexual glands play an important role in the quality of the semen produced and their ultrasound evaluation allows the identification of abnormalities that may compromise the quality of the seminal plasma, generating unsatisfactory results in the fertilizing capacity of the sperm (Ribeiro et al., 2017).

In wild animals, data on ultrasound images of the accessory sexual glands are scarce. Most of the information regarding reproductive ultrasound focuses on the evaluation of the male gonad, as previously described. For some species, we found descriptions related to the ultrasound characteristics of these organs, such as elephants, which presents seminal vesicles (large, anechoic and with a wall containing a distinguishable muscular layer and mucosa), prostate (a pair of glands with three lobes each and connected by a bridge; presence of an anechoic cavity in the African species) and bulbourethral glands (solid appearance with anechoic central cavity) characterized (Hildebrandt et al., 2000).

In the Asian bearcat, only the prostate is found, characterized as a hypoechoic structure, surrounded by a hyperechoic capsule and oval. The position resembles that found in dogs and cats (Lajara et al., 2021). In koalas, in addition to the prostate like those already described, there are the bulbourethral glands presenting hypoechogenic (Larkin et al., 2018). Wojick et al. (2018) describe the accessory sexual glands of aardvarks (*Orycteropus afer*) as being like those of other mammals.

None of the mentioned authors establish a relationship between ultrasound images and the individual's fertilizing capacity, even though the influence of their products on seminal quality is clear. Hildebrandt et al. (2000) highlight the difference between images of glands in prepubertal animals and those at reproductive age, and therefore, it is a tool for analyzing the sexual development of the individuals analyzed.

## Ultrasonography as a tool for determining gender and reproductive stage

Some animal species do not exhibit sexual dimorphism, which directly impacts their reproductive management. This occurs more frequently in bird, reptile and fish species, and less frequently in mammals.

In sturgeon fish, early sexing favors the management of the species in production systems, and ultrasonography is a valid tool since the image of echogenic ovaries differs from the image of hypoechogenic testicles (Masoudifard et al., 2011). In lizards, ultrasonography does not seem to be the most reliable method due to anatomical characteristics of the species, such as scales in the cloaca region or even the isoechogenicity of the hemipenis when compared to the tail muscles (Di Ianni et al., 2014). In contrast, Morris and Alberts (1996) reported successful ultrasound-based sex determination in White-Throated Monitors (*Varanus albigularis*), Gila Monsters (*Heloderma suspectum*), and Beaded Lizards (*H. horridum*). This variability in outcomes emphasizes the necessity for species-specific protocol development and validation. In this context, other diagnostic methods can be used, such as contrast radiography or contrast computed tomography, but it is important to always evaluate the anatomical characteristics of the genders to choose the best technique to be used.

Another aspect that can be analyzed by ultrasound is sexual seasonality. In belugas (*Delphinapterus leucas*), the size of the testicles visualized by ultrasound showed a seasonal variation of 60% between the reproductive and non-reproductive periods. For the morphometric evaluation of the beluga testicle, the cubic fixed effect model was used (Richard et al., 2017). In dolphins (*Tursiops truncatus aduncas*), the ultrasound examination proved to be easy to perform, with a lobulated aspect observed in the testicle of older animals, while in young animals it was cylindrical. In addition, an increase in echogenicity was observed in older animals, which leads to the possibility of characterizing fertility by ultrasound examination in this species (Brook et al., 2000).

## Perspectives

Advances in imaging diagnostic equipment technology bring with them an exciting prospect of obtaining important data for animal reproduction. Increasingly accurate images facilitate the evaluation and standardization of information. In addition, new technologies allow access to previously unknown information. The improvement in the use of doppler in conjunction with ultrasound is a clear example of this evolution.

In domestic animals, it is possible to correlate a male's reproductive capacity with characteristics of gonadal blood flow. Parameters such as resistivity and pulsatility index, as well as velocimetric data such as peak systolic velocity and end diastole velocity, have proven to be good markers for fertility in donkeys (Abdelnaby et al., 2021). However, breed variations should be considered when evaluating Doppler velocimetric indices. In bulls, although further studies are needed, variations were observed between the Brangus, Nelore, and Hereford breeds, demonstrating the need to standardize the data obtained (Barca et al., 2018).

The indices obtained by Doppler evaluation can characterize the blood flow of a given organ. In the case of male gonads, it has been demonstrated that a decrease in blood flow can cause apoptosis of sperm precursor cells, thus reducing the individual's fertility (Bergh et al., 2001). Therefore, it is imperative to know the normal indices of the species and breed analyzed to obtain appropriate conclusions.

Ultrasound of the male reproductive system can also contribute to the identification and characterization of testicular diseases that may be related to decreased fertility. In this context, the association between data obtained using elastography appears to be of great diagnostic value for human patients (Cantisani et al., 2021). The characterization of elastography of healthy testicular tissue also contributes to the standardization of changes. Thus, the exam can be used to reflect the relative hardness of healthy testicular tissue (Chen et al., 2020). These data open perspectives for the use of elastography as a testicular evaluation method, including sperm production capacity.

## Final considerations

Reproductive analysis of wild species is necessary to ensure their survival. Several wild species are at imminent risk of extinction and the promotion of reproductive techniques can help reverse this situation. Analyzing the publications related to the subject we can see that the great interest seems to be focused on the knowledge and manipulation of the reproductive physiology of the female, with diverse investigations both morphological and physiological.

Knowledge about male physiology has focused on seminal aspects, but hormonal and ultrasound assessments can be correlated with seminal quality, bringing a new focus to the knowledge of its physiology and consequently new methods that guarantee the success of reproductive biotechnologies in a greater number of species.

New modalities such as the use of doppler appear to add knowledge about reproductive physiology and other investigations should be carried out to establish reliable parameters related to the production of male gametes. As in domestic species, knowing the variations between wild animals can help to elucidate obstacles in assisted reproduction protocols.

## Data Availability

No research data was used.
